# Molecular analyses identify hybridization‐mediated nuclear evolution in newly discovered fungal hybrids

**DOI:** 10.1002/ece3.5238

**Published:** 2019-05-09

**Authors:** Fabiano Sillo, Paolo Gonthier, Blakey Lockman, Takao Kasuga, Matteo Garbelotto

**Affiliations:** ^1^ Department of Agricultural, Forest and Food Sciences (DISAFA) University of Torino Grugliasco (TO) Italy; ^2^ Pacific Northwest Region, State and Private Forestry USDA Forest Service Portland Oregon; ^3^ Crops Pathology and Genetics Research Unit USDA Agricultural Research Service Davis California; ^4^ Department of Environmental Science, Policy and Management, Forest Pathology and Mycology Laboratory University of California, Berkeley Berkeley California

**Keywords:** forest pathogen, *Heterobasidion*, hybridization, rapid evolution

## Abstract

Hybridization may be a major driver in the evolution of plant pathogens. In a high elevation Alpine larch stand in Montana, a novel hybrid fungal pathogen of trees originating from the mating of *Heterobasidion irregulare* with *H. occidentale* has been recently discovered. In this study, sequence analyses of one mitochondrial and four nuclear loci from 11 *Heterobasidion* genotypes collected in the same Alpine larch stand indicated that hybridization has increased allelic diversity by generating novel polymorphisms unreported in either parental species. Sequence data and ploidy analysis through flow cytometry confirmed that heterokaryotic (*n* + *n*) genotypes were not first‐generation hybrids, but were the result of multiple backcrosses, indicating hybrids are fertile. Additionally, all admixed genotypes possessed the *H. occidentale* mitochondrion, indicating that the hybrid progeny may have been backcrossing mostly with *H. occidentale*. Based on reticulate phylogenetic network analysis by PhyloNet, Bayesian assignment, and ordination tests, alleles can be defined as *H. irregulare*‐like or *H. occidentale*‐like. *H. irregulare*‐like alleles are clearly distinct from all known *H. irregulare* alleles and are derived from the admixing of both *Heterobasidion* species. Instead, all but one *H. occidentale* alleles found in hybrids, although novel, were not clearly distinct from alleles found in the parental *H. occidentale* population. This discovery demonstrates that Alpine larch can be a universal host favouring the interspecific hybridization between *H. irregulare* and *H. occidentale* and the hybridization‐mediated evolution of a nucleus, derived from *H. irregulare* parental species but clearly distinct from it.

## 1 INTRODUCTION

Increased globalization and world trade have resulted in a rise in the number of novel pathosystems, that is, plant diseases caused by new plant pathogens × host species combinations (Depotter, Seidl, Wood, & Thomma, 2016; Stukenbrock, 2016a, 2016b). The long‐distance movement of tree pathogens (Wingfield, Slippers, Roux, & Wingfield, 2001), the long‐distance movement of tree hosts that are planted in “exotic” sites (Burgess & Wingfield, 2016), or restoration using plant stock infested by pathogens (Garbelotto, Frankel & Scanu, 2018; Sims, Tjosvold, Chambers, & Garbelotto, 2019) are all mechanisms known to have generated new pathosystems. The same drivers above are also known to be responsible for the recent comingling of allopatric pathogens or of pathogens with differential host preference, until recently distinctively characterized by nonoverlapping ranges. The movement of pathogenic species with nonoverlapping geographic ranges is particularly relevant because allopatric species are often reported to have maintained the ability to successfully mate with congeneric ones, facilitating the generation of interspecific hybrids (Kohn, 2005).

Indeed, a crucial role in the generation of novel plant pathogens has been ascribed to hybridization between species, a process in which the combination of two or more genomes in a single organism can lead to rapid adaptive evolution (Brasier, 2000; Depotter et al., 2016; McDonald & Stukenbrock, 2016; Olson & Stenlid, 2002; Schardl & Craven, 2003; Stukenbrock, 2016a, 2016b). Some of the more interesting examples of microbial hybridization include tree pathogens, for example, *Phytophthora × alni* (Brasier, Cooke, & Duncan, 1999), *Melampsora × columbiana* (Newcombe, Stirling, McDonald, & Bradshaw, 2000), and *Ophiostoma novo‐ulmi* (Brasier & Buck, 2001). The evolutionary outcomes of interspecific hybridization among plant pathogens are still far from being clearly deciphered; however, a growing body of evidence supports the notion that plant pathogens may develop greater virulence through rapid evolution mediated by hybridization events (Brasier, 2000; Stukenbrock, 2016a). According to this scenario, hybrids can be either transient, but still acting as ephemeral “genetic bridges” between parental species (Brasier, 2000), or they can be viable, as fit as parental individuals, and repeatedly generated as part of hybrid swarms (Gonthier & Garbelotto, 2011). Hybrid swarms that are not reproductively isolated from parental populations and that include ecologically fit and fertile first‐generation hybrids are bound to represent the first step in a process leading to populations comprising individuals with genomes admixed between the two parental species (Gonthier & Garbelotto, 2011). On the other hand, hybridization followed by reproductive isolation has been reported to contribute to rapid speciation in yeast (Leducq et al., 2016), and the same has been hypothesized to occur in some filamentous fungi (Gladieux et al., 2014; Kohn, 2005) and in fungus‐like oomycetes (Schardl & Craven, 2003).

Fungi belonging to the genus *Heterobasidion* (Basidiomycota; Russulales) are among the most damaging pathogens to conifers in temperate regions of the Northern Hemisphere (Garbelotto & Gonthier, 2013). Based on the extensive research available, the *Heterobasidion annosum* species complex is known to comprise the two species *H. irregulare* Garbel. & Otrosina and *H. occidentale* Otrosina & Garbel. in North America, and the three species *H. abietinum* Niemelä & Korhonen, *H. annosum* (Fr.) Bref., and *H. parviporum* Niemelä & Korhonen in Eurasia (Garbelotto & Gonthier, 2013). In North America, *H. irregulare* generally attacks pines and junipers (*Juniperus* spp), while the host range of *H. occidentale* comprises *Abies*, *Picea*, *Tsuga*, *Pseudotsuga*, *Sequoia,* and *Sequoiadendron* (Garbelotto & Gonthier, 2013). *Heterobasidion irregulare* is present throughout North America, whereas *H. occidentale* is only present in Western North America (Garbelotto & Gonthier, 2013). Even when both species coexist in the same region, they are often found in different stands due to their different host preference. When they are found in the same site, they are normally partitioned on different hosts; their true comingling appears to be closely associated with the creation of stumps through logging, a practice that has allowed for the establishment of both species on the same substrate (Garbelotto & Gonthier, 2013). Primary infection and colonization of new forest stands is in fact affected by basidiospores on fresh woody surfaces, such as stumps (Rishbeth, 1959). Basidiospores germinate and the fungus saprobically colonizes the stump including its root system, as well as the roots systems of adjacent individuals, thus infecting neighboring standing trees and more stumps (Garbelotto & Gonthier, 2013). As a result of root‐to‐root secondary infection, *Heterobasidion*‐induced tree mortality appears in groups known as root disease centers that progressively expand in time (Garbelotto & Gonthier, 2013). Human activities have favored the spread of the fungus not only by creating the primary infection substrate, that is, fresh stumps, but also by excluding fires favoring a change in tree species composition and by decreasing/arresting timber harvest operations, thus increasing stand density, which favor tree‐to‐tree contagion.

*Heterobasidion* species are known to retain a certain degree of interfertility. In vitro experiments have indicated the rate of observed interfertility ranges about 5%–98% depending on the species combinations (Garbelotto & Gonthier, 2013; Harrington, Worrall, & Rizzo, 1989; Korhonen & Stenlid, 1998). Hybridization processes in nature have also been documented to occur between pairs of taxa within the species complex (Garbelotto & Gonthier, 2013). The generation of hybrids in *Heterobasidion* spp. in nature, as in the majority of basidiomycetous fungi, can be achieved by plasmogamy of two haploid (*n*) mycelia of interfertile species, which can generate a heterokaryotic mycelium (*n* + *n*) characterized by the co‐occurrence of haploid nuclei from both parental species in the same cell. Since karyogamy is delayed, the generated heterokaryotic mycelium often represents the main growth phase of the hybrid isolate. Only once it is well established on a substrate and when environmental conditions are favorable, the fruiting bodies, in which karyogamy and meiosis occur, may be produced. Meiosis produces haploid (*n*) basidiospores responsible for the infection of new stumps.

In 1996, a natural hybrid genotype between *H. irregulare* and *H. occidentale* was found on a ponderosa pine (*Pinus ponderosa* Laws.) stump in California and in adjoining western juniper (*Juniperus occidentalis* Hook.) and ponderosa pine trees (Garbelotto, Ratcliff, Bruns, Cobb, & Otrosina, 1996). Both parental species were also isolated from the same ponderosa stump. The hybrid was regarded as a first‐generation hybrid due to the presence of both *H. irregulare* and *H. occidentale* isozyme alleles at each of 10 loci (Garbelotto et al., 1996). Surprisingly, later experimental evidence suggested that F1 *Heterobasidion* hybrids may be diploids (2*n*) rather than heterokaryons (*n* + *n* ploidy), indicating that the first step of hybridization affected ploidy (Garbelotto, Gonthier, Linzer, Nicolotti, & Otrosina, 2004), as reported for many plant and animal hybrids (Mallet, 2007). The discovery of both parental species in many stumps across California (Otrosina, Chase, & Cobb, 1992) and of a large hybrid genotype in a stump (Garbelotto et al., 1996) has led to the hypothesis that *H. irregulare* × *H. occidentale* hybridization may require either a universal host or a common substrate where both *Heterobasidion* species can thrive (Garbelotto et al., 1996). The hypothesis that a novel “common” substrate or host may be necessary for hybridization to occur was further reinforced by the finding that fitness of natural and artificial *Heterobasidion* hybrids is reduced on hosts preferred by each parental species (Garbelotto, Gonthier, & Nicolotti, 2007). In California, simultaneous colonization of ponderosa pine stumps by both species (Garbelotto et al., 1996) may have favored interspecific mating and hybridization since commercial logging has been practiced. However, solid evidence of ancient horizontal gene transfer between *H. irregulare* and *H. occidentale* at the continental level in North America (Linzer et al., 2008) suggests that hybridization must have predated the era of commercial logging (e.g., the mid 1800s) and must have occurred in the absence of stumps.

Almost 15 years after the first discovery of a natural hybrid *Heterobasidion* genotype in North America, a *H. irregulare* × *H. occidentale* hybrid genotype was discovered in a mortality center of Alpine larch (*Larix lyalli* Parl.) in the Bitterroot Mountains, south of Darby, Montana (Lockman, Mascheretti, Schechter, & Garbelotto, 2014). This second report of a hybrid between *H. irregulare* and *H. occidentale* suggested that Alpine larch may be a host for both *Heterobasidion* species, as described for naturally infected ponderosa pine stumps in California and for artificially inoculated Sitka spruce (*Picea sitchensis* [Bong.] Carr.) seedlings (Garbelotto et al., 2007). Because no logging has occurred at this high elevation site, the finding potentially provided an opportunity to study natural interspecific hybridization in *Heterobasidion* in North America independent of direct anthropogenic effects. Additionally, if indeed hybridization has been ongoing in these high elevation forests at a significant rate, the study of naturally formed genotypes with admixed genomes may allow to understand the evolutionary implications of hybridization, a topic that has received relatively little attention for the entire fungal kingdom.

This study describes the result of a second more exhaustive sampling of two mortality centers in the same infested Alpine larch stand where the original Lockman et al. (2014) finding had occurred. Sampling included direct isolation of *Heterobasidion* genotypes from infected wood, fruiting bodies, and airspora collected on woody spore traps. Sequence analysis of one mitochondrial and four nuclear loci was then performed on all *Heterobasidion* genotypes obtained through such sampling scheme.

The aims of this study were as follows:

- To collect multiple genotypes and determine their genetic *makeup* (e.g., pure vs. admixed nuclei and mitochondrial type);
- To determine which genomic makeup may be dominant in the area;
- To genetically characterize any admixed genotype and determine the level and parental origin of the admixture;
- To determine whether hybridization may have resulted in an increase in genetic variability.

## 2 MATERIALS AND METHODS

### 2.1 Study site and sampling

In September 2014, two distinct root disease centers were sampled in a stand of the Bitterroot Mountains, on the shores of Gem Lake, south of Darby, Montana (elev. 2,530 m; Lat. 45.893528°, Long. −114.278322°). One site was the same where the first hybrid *Heterobasidion* fruiting body had been original collected (Lockman et al., 2014), the second was adjacent to the first, but clearly separated from it by a treeless buffer, approximately 100 m wide. Each site corresponded to a classical *Heterobasidion* root disease center (Garbelotto & Gonthier, 2013) characterized by dead, dying, and symptomatic larch trees, roughly circular in shape, and with a diameter of about 50 m each.

**Figure 1:**
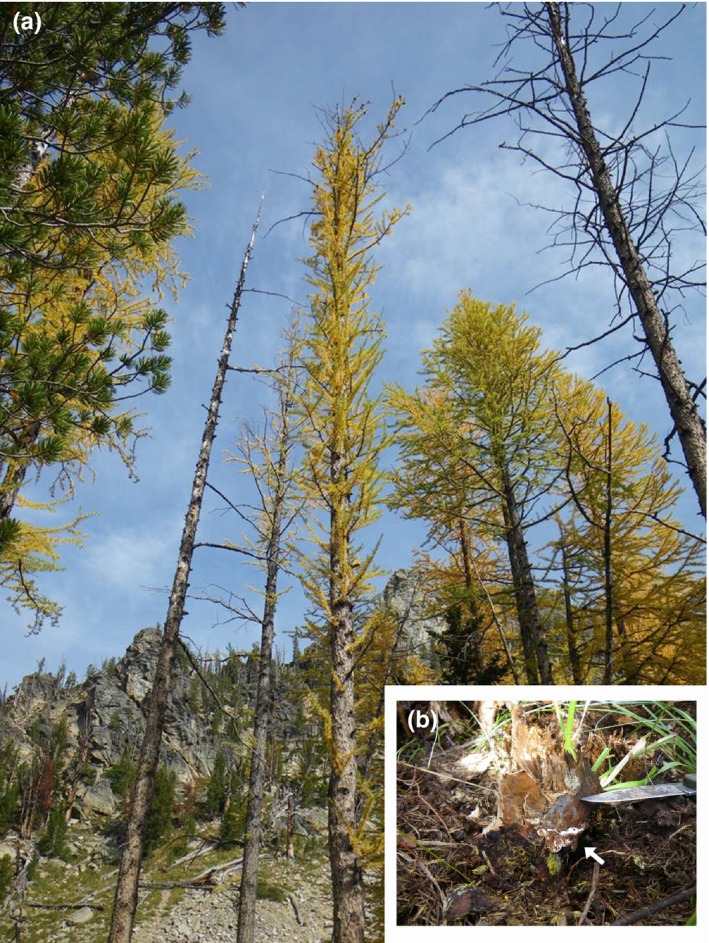
Alpine larches (*Larix lyalli*) in the Bitterroot Mountains (Montana, USA) showing disease symptoms (a) related to the infection by *Heterobasidion* spp. In (b), *Heterobasidion* spp. fruit body (indicated by a white arrow) developed at the base of a fallen tree

In each root disease center, several adjacent Alpine larches (*L. lyallii*) were either dead or displayed thin crowns (Figure 1). The stand is mature, and in the two study sites, 79% of trees were Alpine larches, 17% were whitebark pines (*Pinus albicaulis* Engelm.), and 4% were subalpine firs (*Abies lasiocarpa* [Hooker] Nuttall), but only larches were symptomatic. Approximately 43% of the larches were dead, 22% were in a stage of advanced decline, and 35% were healthy. The stand immediately below the one surveyed was characterized by an overwhelming majority of subalpine firs.

In each disease center, isolations were performed directly from fruiting bodies or wood symptomatic for decay, and by subculturing *Heterobasidion* colonies growing on woody traps. A total of fifteen traps per site were placed in groups of three at 10‐m intervals along a 50‐m‐long linear transect and exposed to the air for a total sampling period of 24 hr.

In order to isolate fungal mycelia from wood or fruiting bodies, fragments of wood, and fruiting bodies 25 mm^2^ in size were surface sterilized in laboratory by using 70% Ethanol and placed on 9‐cm Petri dishes containing MEA (dilute Malt Extract Agar; 2 g malt extract, 10 g glucose, 2 g peptone, 20 g agar, 1 L distilled water, amended with 0.05 g/L ampicillin). Purification of isolates from contaminants was performed under a dissecting microscope (20× magnification) by transferring putative mycelium of *Heterobasidion* spp. from the original Petri dishes onto sterile Petri dishes filled with MEA.

In order to collect isolates from aerial spores, spores of *Heterobasidion* spp. were trapped using the wood‐disk exposure method as previously described (Gonthier, Nicolotti, Linzer, Guglielmo, & Garbelotto, 2007). Briefly, wood disks of 11–13 cm diameter were individually placed in 15‐cm Petri dishes containing sterile filter papers dampened with 3.5 ml of sterile water, to prevent drying during exposure. A total of 45 open Petri dishes were placed along three transects with five trapping points on the ground at 10 m from one another, each including three woody traps placed at 1 m from one another at the vertices of an imaginary triangle, for a total of five trapping points per transect. After a 24‐hr exposure, filter papers were replaced and dampened again with 3 ml of sterile water. Wood disks were sprayed with a benomyl solution (0.010 g benomyl, 500 μL methanol, and 1 L sterile water) and incubated at 24°C for 15 days. Isolations were made under a dissecting microscope (20× magnification) by transferring colonies of *Heterobasidion* spp. in its conidial stage (*Spiniger*) onto Petri dishes filled with MEA and amended with 0.3 g/L streptomycin (Kuhlman & Hendrix, 1962). Spore load was calculated as spores/m^2^ *per* hour (spores/m^2^ h) according to Gonthier, Garbelotto, and Nicolotti (2005). All obtained isolates were subsequently grown at 24°C on 6‐cm Petri dishes filled with MEA.

### 2.2 DNA content analysis by flow cytometry

DNA contents were measured by flow cytometry for five *Heterobasidion* isolates from wood samples randomly chosen. The isolate (ID: Awr400) identified in 1996 by Garbelotto et al. (1996) as a first‐generation hybrid between *H. irregulare* and *H. occidentale* was included in the analysis as a putative diploid control. *Heterobasidion* isolates were taken from MEA slants and grown in test tubes containing 5 ml filter‐sterilized 5% clarified V8 broth (Englander & Roth, 1980) for 3 days at 21°C. The mycelium was checked for the absence of conidia under the dissecting microscope, then harvested, and washed three times with sterile water. *Arabidopsis thaliana* Col‐0 and *Aspergillus fumigatus* CEA10 (FGSC A1163) were chosen as the internal DNA reference standards with a genome size of 1C = 157 Mb (Dolezel & Bartos, 2005) and 1C = 29.7 Mb (Veselska, Svoboda, Ruzickova, & Kolarik, 2014), respectively. A modified protocol derived from Bertier, Leus, and D'hondt, de Cock, and Höfte (2013) was then followed. In brief, extraction of nuclei was done using the Cystain PI absolute P kit (Sysmex America Inc. cytometry@sysmex.com). For each sample, approximately five flower buds of *A. thaliana* or 3‐day‐old *A. fumigatus* mycelium grown in the clarified V8 broth and a small amount of *Heterobasidion* mycelium were cochopped with a razor blade (Gillette) in a Petri dish containing 500 μl extraction buffer. After chopping, the suspension was filtered through a 10 μm filter (CellTrics, Sysmex America Inc.) and 2 ml of a Propidium Iodide staining solution was added. The samples were incubated for 60 min in the dark at 4°C. Measurements were done on a Becton Dickinson FACScan equipped with a 488 nm laser and a 585/42 nm band‐pass filter. The data were analyzed using FlowJo v.10 (https://www.flowjo.com/solutions/flowjo), and DNA content was subsequently inferred using a quadratic regression with the ratios between the peak positions of the *Heterobasidion* sample and the two standards.

### 2.3 DNA extraction and PCR conditions

For DNA extraction, isolates were grown in 250‐ml flasks filled with malt extract broth (2% w/v), at room temperature and in the dark for 10 days. Mycelia from isolates were collected through a vacuum pump and dry lyophilized. About 100 mg of lyophilized fungal material per isolate were homogenized through the use of glass beads (3 mm and 5 mm of diameter) in a FastPrep FP120 Cell Disrupter (QBioGene). DNA extraction was performed by using the E.Z.N.A.™ Stool DNA Isolation Kit (Omega Bio‐Tek).

Five genomic loci were selected as markers to conduct the phylogenetic analysis. These five loci have proven to be reliable for phylogenetic studies of the genus *Heterobasidion*, and have all been widely used in the past (Chen, Cui, Zhou, Korhonen, & Dai, 2015; Dalman, Olson, & Stenlid, 2010; Johannesson & Stenlid, 2003; Linzer et al., 2008). The markers were the mitochondrial ATPase subunit 6 (*atp6*), the glyceraldehyde‐3‐phosphate dehydrogenase (*gpd*), the RNA polymerase II 2nd largest subunit (*RPB2*), the translation elongation factor 1‐alpha (*EF‐1α*), and the nuclear ribosomal internal transcribed spacer (ITS). Primers used for the amplification of *atp6* were 5′‐TAATTCTANWGCATCTTTAATRTA‐3′ (ATP6‐2; forward) and 5′‐TCTCCTTTAGAACAATTTGA‐3′ (ATP6‐3; reverse) (Kretzer & Bruns, 1999); for *gpd* were 5′‐AGCCTCTGCCCAYTTGAARG‐3′ (GPD1; forward) and 5′‐RTANCCCCAYTCRTTRTCRTACCA‐3′ (Glyceraldehyde 3‐phosphate dehydrogenase R; reverse) (Linzer et al., 2008); for *RPB2* were 5′‐GAYGAYMGWGATCAYTTYGG‐ 3′ (fRPB2‐5F; forward) and 5′‐CCCATRGCTTGYTTRCCCAT‐3′ (fRPB2‐7cR; reverse) (Matheny, 2005); for *EF‐1α* were 5′‐TCAACGTGGTCGGTGAGCAGGTA‐3′ (forward) and 5′‐AAGTCACGATGTCCAGGAGCATC‐3′ (reverse) (Johannesson & Stenlid, 2003); for ITS were 5′‐CTTGGTCATTTAGAGGAAGTAA‐3′ (ITS1f; forward) and 5′‐TCCTCCGCTTATTGATATGC‐3′ (ITS4; reverse) (Gardes & Bruns, 1993). PCRs were performed in the following 25 µl reaction mixture: 5× buffer, 0.2 mM dNTPs, 1.25 U/µl of GoTaq polymerase (Promega Corp.), 2.0 mM MgCl_2_ (Invitrogen Corp.), 0.50 µM each nondegenerate primer or 0.64 µM each degenerate primer (Table 2), and approximately 20 ng of DNA. The Promega GoTaq polymerase used in the study is considered as one of the best non‐proof‐reading high‐quality and high‐performance polymerases on the market (www.promega.com). Schneider et al. (2013) reported that this enzyme showed an error rate of 0.15 SNPs/kb (Schneider et al., 2013). The PCR programs used for *atp6, gpd, EF‐1α,* and ITS were as follows: 95°C for 3 min, followed by 35 cycles of 95°C for 40 s, different annealing temperature (50°C for *atp6*, 52°C for *gpd*, 66°C for *EF‐1α*, 53°C for ITS) for 55 s, 72°C for 55 s, and an extension step at 72°C for 7 min. The PCR program for *RPB2* was as follows: 94°C for 2 min, followed by 10 cycles at 94°C for 40 s, 60°C for 40 s, 72°C for 2 min, followed by 37 cycles at 94°C for 45 s, 55°C for 1.5 min and 72°C for 2 min, and an extension step at 72°C for 10 min.

### 2.4 Cloning of *EF‐1 α* and ITS alleles

In order to obtain haplotype phases of sequences of heterokaryotic isolates, avoiding high heterozygous sequences, sequences showing more than two heterozygous sites, that is, a single copy gene (*EF‐1α*) and a multi‐copy locus (ITS), were cloned. In detail, two clone libraries *per* isolate were generated starting from PCR amplicons of the two loci, for a total of 12 libraries. PCR products from amplification of *EF‐1α* and ITS were purified through the use of the enzyme ExoSAP‐IT (Affymetrix), cloned into plasmids using the TOPO TA Cloning kit (Invitrogen) according to the manufacturer's instructions, and transformed into *Escherichia coli* competent cells. Positive colonies from each library were amplified using T7 (5′‐TAATACGACTCACTATAGGG‐3′; forward) and M13 (5′‐GTAAAACGACGGCCAGT‐3′; reverse) primers and amplicons were visualized on 1.5% agarose gel. For each library, eight positive colonies were Sanger‐sequenced in house at the Forest Pathology and Mycology Laboratory (Berkeley, USA) and at BMR Genomics S.R.L. (Padua, Italy), for a total of 48 sequences for *EF‐1α* and 48 sequences for ITS.

### 2.5 Sequencing and phylogenetic analyses

The purified PCR products of *atp6, gpd,* and *RPB2* amplicons were Sanger‐sequenced in house at the Forest Pathology and Mycology Laboratory (Berkeley, USA) and at BMR Genomics S.R.L. (Padua, Italy). The 96 purified cloned products of *EF‐1α* and ITS were also Sanger‐sequenced in the same laboratories. All amplicons were forward and reverse sequenced with the related primers, and consensus sequences were generated by using the Geneious software, version 9.0.5 (Biomatters, Ltd). Chromatograms of each sequence were analyzed by using both Geneious and SnapGene® Viewer. The minimum acceptable *Phred* score considered *per* base was 20. For the two not‐cloned loci, that is, *gpd* and *RPB2,* ambiguous bases showing two overlapping peaks with equal signal intensity were assigned manually based on the allele frequencies of the putative parental species, that is, *H. irregulare* and *H. occidentale*. When a SNP in the heterozygotic sequence could not be assigned as being derived from one of the two parents, it was marked as the same ambiguous nucleotide on both alleles. This process allowed to extract with confidence the two homozygotic alleles, each derived from one of the two parents, from uncloned heterozygotic sequences.

For each locus, a multiple sequence alignment was built using the ClustalW algorithm embedded in MEGA version 6 (Tamura, Stecher, Peterson, Filipski, & Kumar, 2013). In order to understand the relationships among haplotypes (alleles), phylogenetic trees were generated using the Maximum Likelihood (ML) method (Substitutions Type: nucleotide; Model: Jukes‐Cantor model, as the simplest substitution model), with the MEGA v. 6 software. Bootstrap analyses were carried out on the basis of 1,000 resamplings of the sequence alignment. Nodes were considered as supported if bootstrap values were greater than 50%. A total of 410 *Heterobasidion* spp. nucleotide sequences deposited in GenBank were included in the phylogenetic analysis. In detail, 131 available sequences were used to infer phylogenetic relationship among *EF‐1α* sequences, 44 for *atp6*, 116 for ITS, 89 for *gpd*, and 30 for *RPB2*. In addition, an alignment of concatenated loci *EF‐1α, gpd* was performed by including new generated homozygotic sequences and 26 concatenated sequences obtained by concatenation of available sequences in GenBank (Table S1). Available sequences of the two North American *Heterobasidion* species, *H. irregulare* and *H. occidentale*, used in the analysis, include alleles of isolates from Alabama, California, Iowa, Mississippi, Montana, South Carolina and Washington in the USA, from Ontario and Quebec in Canada, and from the State of Mexico, in the Mexican Republic. Representative sequences generated by this study were deposited in Dryad Digital Repository (https://doi.org/10.5061/dryad.6t7sh85). The Tajima's relative rate test embedded in MEGA v.6 was performed on triplets of concatenated *EF‐1α* and *gpd* sequences, to test whether the molecular clock hypothesis should be rejected or not. In detail, each concatenated allele was compared with concatenated sequences of *H. irregulare* (isolate Conk3) and *H. occidentale* (isolate PFC 5,357), using a sequence of *H. annosum* s.s. (isolate 417P) as an out‐group for the test.

### 2.6 Multidimensional scaling, Bayesian and PhyloNet analyses

A distance matrix from the alignments of the two concatenated loci *EF‐1α* and *gpd* was obtained using the MEGA v. 6 software on a dataset including sequences from the 11 isolates obtained in this study, supplemented with sequences from 26 reference haplotypes, obtained from 18 genotypes of *H. irregulare*, 5 genotypes of *H. occidentale*, and 3 genotypes *H. annosum* s.s. In order to perform the concatenation, homozygotic alleles extracted from uncloned heterozygotic *gpd* sequences were used. Each SNP was assigned as being derived from either parent based on allelic variation present in parental reference sequence, as explained above. The distance matrix obtained in MEGA v. 6 was used in GenAlex v. 6.501 (Peakall & Smouse, 2006) to perform a principal coordinates analysis (PCoA) in order to visualize similarities/dissimilarities within the dataset. Bayesian analysis of population structure using STRUCTURE v. 2.3.4 (Pritchard, Stephens, & Donnelly, 2000) was also performed on the concatenated sequence alignments, including 17 concatenated sequences from this study and 23 out of 26 sequences from the same reference genotypes listed above (*H. annosum* s.s. data were excluded). As input file for STRUCTURE analysis and in accordance with instructions provided by the STRUCTURE manual for analysis of sequence data, sequence data were number‐coded as single nucleotide polymorphism (SNP) data, that is, each haplotype include a single locus (numeric data) with *n* alleles corresponding to polymorphisms. successive K values (number of populations/groups/species) from 2 to 10 were used to obtain the distinct clusters and to estimate number of subpopulations. Twenty runs each for *K* = 2–10 with 1,000,000 MCMC repetitions after a burn‐in period of 50,000 repetitions were performed, with the parameter set as “Admixture Model” and “Allele frequencies independent,” and without any prior information of the origin of individual samples. The determination of Δ*K* based on the highest likelihood of the data (LnP(*D*)) was used to infer the *K* value best representing the observed data under the implemented model (Evanno, Regnaut, & Goudet, 2005) using Structure Harvester Web v0.6.94 (Earl & vonHoldt, 2012).

In order to test sequences from Montana Larch isolates for signs of hybridization and introgression events, the software PhyloNet (Than, Ruths, & Nakhleh, 2008) was used. In detail, concatenated sequences of *EF‐1α* and *gpd* from reference accessions of *H. irregulare*, *H. annosum* s.s., and *H. occidentale,* and sequences from the heterokaryotic Montana isolates and from the hybrid spore III2B collected in this study were merged as five consensus sequences using the EMBOSS command “*consambig*” (http://www.bioinformatics.nl/cgi-bin/emboss/consambig). These five consensus sequences represented alleles from the three species, that is, *H. irregulare*, *H. annosum* s.s., and *H. occidentale,* and the two groups of novel alleles from the studied isolates. These sequences were included in a NEXUS file. Bayesian Markov Chain Monte Carlo (MCMC) posterior estimation of phylogenetic networks and sequence trees were generated by PhyloNet through the command *MCMC_SEQ* with parameters –cl (chain length) set at 10,000,000 and –bl (iterations in burn‐in period) set at 2,000,000. Maximum number of reticulations was set up at 5. The substitution model was the JC69 model (default). Generated trees were used as inputs for the command *MCMC_GT* with default parameters in order to perform the estimations of parental genome contributions (inheritance probabilities) in putative hybrid sequences. Trees were visualized by using Dendroscope v 3.5.9 (Huson & Scornavacca, 2012). Hybridization network tree was built using the Autumn algorithm (Huson & Linz, 2018) embedded in Dendroscope.

## 3 RESULTS

### 3.1 Sampling and obtaining of fungal isolates

After sampling and direct isolation, a total of seven heterokaryotic (ploidy = *n* + *n*) isolates were obtained from wood samples and one heterokaryotic isolate was obtained from a fruiting body. Six of them were used for molecular analyses (Table 1). Five homokaryotic (ploidy = *n*) colonies were isolated from spore traps and also used in the molecular analyses; four were pure *H. occidentale*, and one was admixed (Table 1). The spore load resulted as high as 18 spores/m^2^ h. All isolates from wood samples, fruiting bodies and from spores used in the molecular analyses were deposited at the Mycotheca Universitatis Taurinensis (MUT). Their related accession numbers are listed in Table 1.

**Table 1:** Summary of *Heterobasidion* isolates

ID_isolate #	Isolated from	Site	MUT accession N.	Tree or trap number
II2B	Spore trap	Site 2	MUT00005886	2B
II1A	Spore trap	Site 2	MUT00005885	1A
II5A	Spore trap	Site 2	MUT00005884	5A
II4C	Spore trap	Site 2	MUT00005883	4C
III2B	Spore trap	Site 1	MUT00005882	2B
MH3001	Alpine larch root	Site 1	MUT00005896	2
MH3002^a^	Alpine larch root	Site 1	NA	2
MH3003	Alpine larch root	Site 2	MUT00005915	4
MH3004^a^	Alpine larch root	Site 1	NA	4
MH3005	Alpine larch root	Site 1	MUT00005993	1
MH3006	Fruiting body	Site 1	MUT00005892	1
MH3007	Alpine larch root	Site 1	MUT00005891	1
MH3011	Alpine larch root	Site 1	MUT00005890	3

Abbreviation: NA, not available.

a Only used for flow cytometry; not used for molecular analysis.

### 3.2 Chromosome analysis by flow cytometry

**Figure 2:**
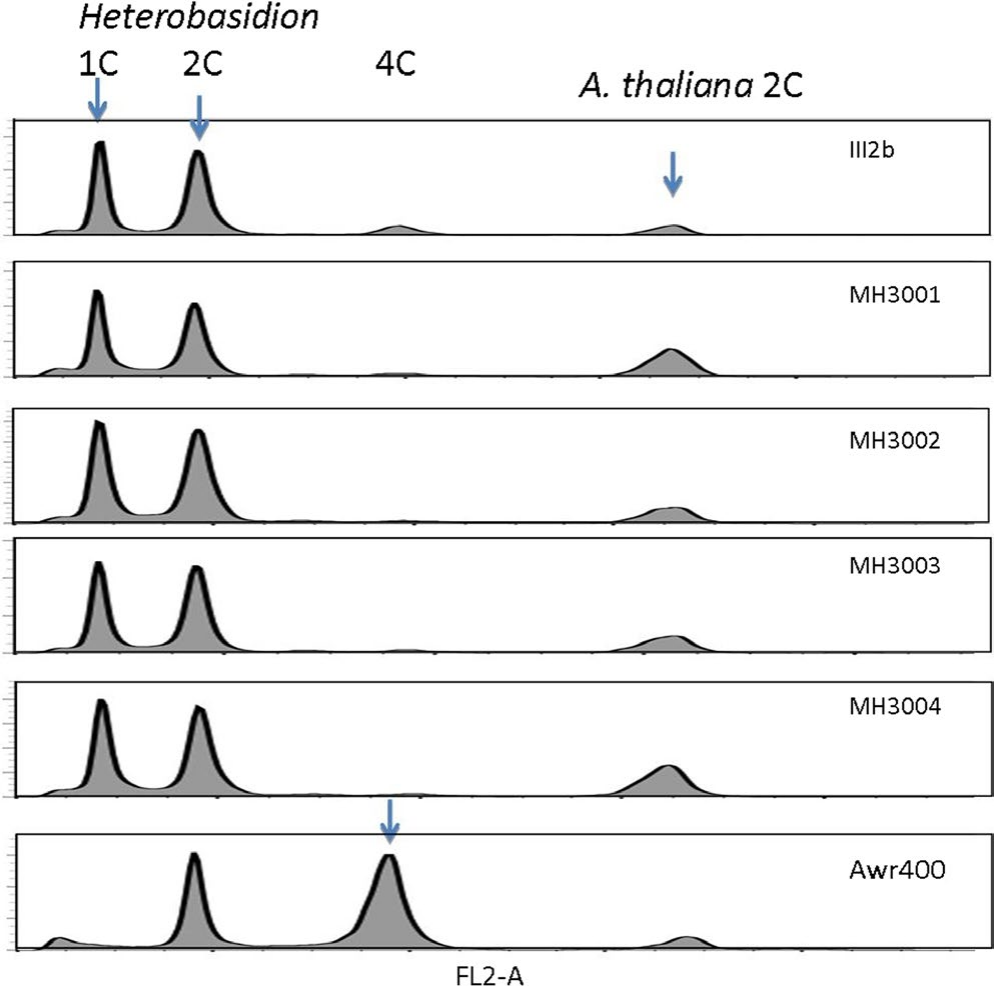
Flow cytometry histograms of six isolates. *Arabidopsis thaliana* was used as an internal standard (2C = 313 Mbp). The nuclei of samples were stained by propidium iodine. There are two main peaks in each panel corresponding to the G1‐ and the G2‐phase nuclei. Haploid DNA amounts (C‐values) for *Heterobasidion irregulare* and *A. thaliana*. *X*‐axis shows FL2‐A intensity of fluorescence measured through a 585/42 nm band‐pass filter in arbitrary unit, whereas *y*‐axis indicates number of observed nuclei

Flow cytometry with two size standards, that is, *A. fumigatus* and *A. thaliana* gave a haploid genome estimate of 34.7 Mbp (Figure S1), a result in good agreement with the genome assembly size of *H. irregulare* (33.1 Mbp) (Olson et al., 2012). All five isolates showed two main peaks, with sizes corresponded to 1C (unreplicated haploid genome at G1 cell cycle phase) and 2C (replicated haploid DNA at G2 and M phases; Figure 2). On the other hand, a first‐generation hybrid isolate Awr400 21B, which a previous study suggested may be diploid (Garbelotto et al., 2004), showed two peaks corresponding to 2C and 4C of diploid cells.

### 3.3 Sequence analysis

Sequencing of amplicons resulted in a total of 11 *atp6* sequences for the 6 heterokaryotic and the 5 homokaryotic isolates. After sequence trimming, 445 nucleotides per *atp6* sequence were aligned. No ambiguous bases were detected in this set of sequences. All sequences were identical, showing no SNPs within them (Table 2).

**Table 2:** Alleles observed in the 11 isolates for each locus

Locus	*atp6*	*gpd*	SNPs related to *H. irregulare*	SNPs related to *H. occidentale*	Private SNPs	*RPB2*	SNPs related to *H. irregulare*	SNPs related to *H. occidentale*	Private SNPs	*EF‐1 alpha*	SNPs related to *H. irregulare*	SNPs related to *H. occidentale*	Private SNPs	*ITS*	SNPs related to *H. irregulare*	SNPs related to *H. occidentale*	Private SNPs
Isolate #ID																	
MH3001	*Hocc*	*Hi‐A*	14/19	5/19	0/19	*Hi‐A*	32/40	8/40	0/40	*Hi‐A*	*23/33*	*4/33*	*6/33*	*Hi‐A*	17/26	7/26	2/26
		*Ho‐A*	7/19	12/19	0/19	*Ho‐A*	14/40	26/40	0/40	*Hocc*	–	–	–	*Hi‐B*	16/26	5/26	5/26
														*Hocc*	–	–	–
MH3003	*Hocc*	*Hi‐C*	16/19	3/19	0/19	*Hi‐B*	31/40	9/40	0/40	*Hi‐B*	*21/33*	*6/33*	*6/33*	*Hi‐A*	17/26	7/26	2/26
		*Ho‐C*	5/19	14/19	0/19	*Ho‐B*	16/40	24/40	0/40	*Hocc*	–	–	–	*Hocc*	–	–	–
														*Ho‐A*	0/26	25/26	1/26
MH3005	*Hocc*	*Hi‐D*	15/19	4/19	0/19	*Hi‐A*	32/40	8/40	0/40	*Hi‐A*	*23/33*	*4/33*	*6/33*	*Hi‐B*	16/26	5/26	5/26
		*Ho‐D*	6/19	13/19	0/19	*Ho‐A*	14/40	26/40	0/40	*Hocc*	–	–	–	*Hi‐C*	18/26	5/26	3/26
														*Hocc*	–	–	–
MH3006	*Hocc*	*Hi‐D*	15/19	4/19	0/19	*Hi‐B*	31/40	9/40	0/40	*Hi‐B*	*21/33*	*6/33*	*6/33*	*Hi‐B*	16/26	5/26	5/26
		*Ho‐D*	6/19	13/19	0/19	*Ho‐B*	16/40	24/40	0/40	*Hocc*	–	–	–	*HiXHo‐F*	10/26	13/26	3/26
														*Hocc*	–	–	–
MH3007	*Hocc*	*Hi‐B*	12/19	7/19	0/19	*Hi‐B*	31/40	9/40	0/40	*Hi‐B*	*21/33*	*6/33*	*6/33*	*Hi‐B*	16/26	5/26	5/26
		*Ho‐B*	6/19	13/19	0/19	*Ho‐B*	16/40	24/40	0/40	*Hocc*	–	–	–	*Ho‐B*	0/26	24/26	2/26
														*HiXHo‐F*	10/26	13/26	3/26
														*Hocc*	–	–	–
MH3011	*Hocc*	*Hi‐C*	15/19	4/19	0/19	*Hi‐B*	31/40	9/40	0/40	*Hi‐A*	*23/33*	*4/33*	*6/33*	*Hi‐B*	16/26	5/26	5/26
		*Ho‐C*	5/19	14/19	0/19	*Ho‐B*	16/40	24/40	0/40	*Ho‐A*	*4/33*	*24/33*	*5/33*	*Hi‐D*	16/26	10/26	0/26
														*Ho‐C*	0/26	25/26	1/26
														*Ho‐B*	0/26	23/26	3/26
														*Hocc*	–	–	–
II1A	*Hocc*	*Hocc*	–	–	–	*Hocc*	–	–	–	*Hocc*	–	–	–	*Hocc*	–	–	–
II5A	*Hocc*	*Hocc*	–	–	–	*Hocc*	–	–	–	*Hocc*	–	–	–	*Hocc*	–	–	–
II2B	*Hocc*	*Hocc*	–	–	–	*Hocc*	–	–	–	*Hocc*	–	–	–	*Hocc*	–	–	–
III2B	*Hocc*	*Hocc*	–	–	–	*Hocc*	–	–	–	*Hi‐A*	*23/33*	*4/33*	*6/33*	*Hi‐A*	17/26	7/26	2/26
II4C	*Hocc*	*Hocc*	–	–	–	*Hocc*	–	–	–	*Hocc*	–	–	–	*Hocc*	–	–	–

Note

Multiple alleles were named by using letters. The tags “*Hi*” and “*Ho*” represents novel “*Heterobasidion irregulare*‐like” and “*Heterobasidion occidentale*‐like” alleles, respectively, while the tag “*Hocc*” means “*H. occidentale*” allele. Polymorphisms related and unrelated (private) to putative parental species over total number of detected SNPs (referred to putative parental species) are also indicated.

Eleven *gpd* sequences, one for each isolate, were obtained and in total four different alleles were identified from heterokaryons (ploidy *n* + *n*) while one additional allele was identified from the homokaryons (ploidy *n*). After sequence trimming, 326 nucleotides *per* sequence were aligned. Number of ambiguous bases showing double peaks were 11, 13, 8, 8, 10, and 9 for isolate MH3001, MH3003, MH3005, MH3006, MH3007, and MH3011, respectively. Ambiguous bases represented polymorphisms derived either from *H. irregulare* or from *H. occidentale*; however, each of the four alleles had a unique combination of such polymorphisms. When extracting the two homozygotic alleles from the six uncloned heterozygotic sequences, all polymorphisms could be clearly assigned as being derived from either parent and no ambiguities remained unresolved (Table 2). Additionally, no ambiguous bases were observed for sequences obtained from the five homokaryotic isolates (Table 2 and Figure S2).

Sequencing of locus *RPB2* generated 11 sequences for the 11 isolates and included three different alleles from heterokaryons and a fourth distinct allele from the homokaryons. After sequence trimming, 800 nucleotides *per* sequence were aligned. Number of ambiguous bases showing double peaks were 16, 17, 7, 16, 12, and 6 for isolate MH3001, MH3003, MH3005, MH3006, MH3007, and MH3011, respectively. All ambiguous bases represented polymorphisms derived either from *H. irregulare* or from *H. occidentale*. Again, the extraction of the two homozygotic alleles from the six uncloned heterozygotic sequences allowed to assign each SNP as being derived from either parent and no ambiguities remained unresolved (Table 2 and Figure S3). As for *gpd*, no ambiguous bases were observed in the five identical sequences from the five homokaryotic isolates (Table 2).

After sequence trimming of the *EF‐1α* locus, 361 nucleotides *per* sequence were aligned. Sequences of *EF‐1α* from homokaryotic isolates were all identical to one another, with the exception of sequence of isolate III2B that showed two indels (not affecting frameshift) and four unique but synonymous SNPs unrelated to either *H. occidentale* or *H. irregulare*. Sequenced alleles of cloned *EF‐1α* locus amplicons from the heterokaryotic isolates (eight cloned sequences *per* isolate) resulted in a total of four different alleles. In detail, each isolate showed two alleles at this locus, one clearly derived from and synonymous with *H. occidentale* alleles, and one clearly derived from and synonymous with *H. irregulare* alleles (Table 2).

Sequence trimming of ITS locus resulted in an alignment of 539 nucleotides *per* sequence. Again, sequences of ITS of homokaryotic isolates were all identical to one another, with the exception of sequence of isolate III2B which showed two synonymous SNPs unrelated to SNPs found in both *H. occidentale* and *H. irregulare*. Sequencing of cloned ITS alleles of the heterokaryotic isolates (eight cloned sequences *per* isolate) resulted in a total of eight different alleles (Table 2). Overall mean nucleotide distance within alleles observed in a single isolate ranged from 0.004 (*SD* 0.005, isolate MH3003) to 0.013 (*SD* 0.003, isolate MH3005).

As shown in Table 2, by comparing sequences of hybrids with those of parents, it was possible to identify two significant mechanisms resulting in de novo nucleotide polymorphisms: (a) SNPs were derived from either parent through hybridization creating a new admixed sequence and (b) SNPs were novel and not derived from either parent, suggesting rapid evolution occurring during hybridization but not directly derived by admixing of the parental alleles.

### 3.4 Phylogenetic analyses

Alignments of mitochondrial (*atp6*) and nuclear (*gpd*, *RPB2*, *EF‐1α,* and ITS) gene sequences were used to infer the phylogenetic placement of the 11 *Heterobasidion* genotypes collected from wood samples, fruiting bodies, and spore traps.

Phylogenetic analysis of *atp6* sequences indicated that all 11 isolates exclusively possessed the *H. occidentale* mitochondrial genome (Figure S4). Conversely, trees obtained from heterozygotic *gpd* and *RPB2* sequences, which harbored ambiguous bases, showed heterokaryotic isolates as being placed in an intermediate position in between reference sequences from the putative parental species *H. irregulare* and *H. occidentale* (Figures S5 and S6). However, when ambiguity in alleles was resolved by manual assignment of bases based on parental allele frequencies, the alignment of sequences showed that each heterokaryotic isolate possessed two distinct types of *gpd* and *RPB2* alleles, one clearly related to *H. irregulare* and one clearly related to *H. occidentale* (Figures S2 and S3).

The phylogenetic analysis of *EF‐1α* cloned sequences showed that all heterokaryotic isolates possessed at least one *H. occidentale* allele (Figure S7). The other alleles found in the isolates grouped together as a separate, statistically well supported, *H. irregulare*‐like cluster sister to the *H. irregulare* cluster (Figure S7). In addition, sequences of isolates MH3006 and MH3007 formed a distinct group but nested within the *H. irregulare‐*like cluster (Figure S7). Sequences from homokaryotic genotypes from spores grouped with the *H. occidentale* cluster, with the exception of the sequence of the genotype III2B, which grouped within the *H. irregulare‐*like subcluster and together with the heterokaryotic larch genotypes (Figure S7).

Topology of the tree based on ITS cloned sequences was similar to that obtained with *EF‐1α* sequences, with a statistically well‐supported *H. irregulare*‐like cluster distinct from *H. irregulare* including one allele from each heterokaryotic isolate (Figure S8). However, each heterokaryotic isolate also had at least one *H. occidentale* allele (Figure S8). Three sequences of isolate MH3006 and one sequence of MH3007 did not cluster with the others but remained unresolved between the two large *H. occidentale‐* and *H. irregulare*‐like clusters. (Figure S8).

The average nucleotide variation observed in cloned *EF‐1α* and ITS alleles of *H. irregulare*‐like was significant (approximately 30 SNPs/361 bp for *EF‐1α* and 25 SNPs/539 bp for ITS, corresponding to 83 and 46 SNPs/Kbp, respectively) and much higher than that expected (0.15 SNPs/Kbp) solely from PCR and cloning errors. Furthermore, cloned alleles used in the study were those cloned multiple times not only from a single genotype, but from at least two different genotypes, thus minimizing the likelihood of basing our analyses on artifacts caused by cloning errors.

**Figure 3:**
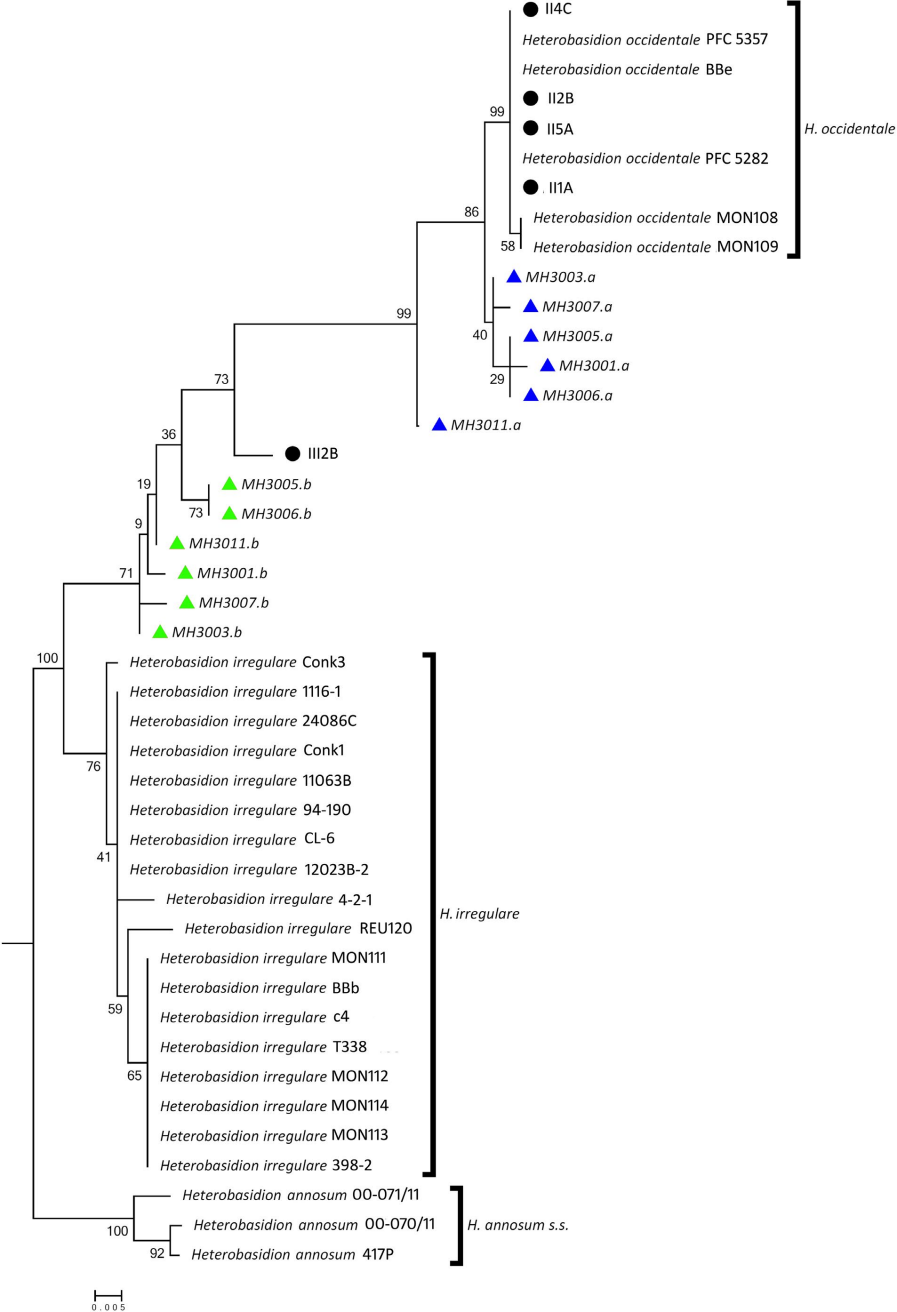
Phylogenetic tree of concatenated *EF‐1α* and *gpd* sequences. Tree is generated by using maximum likelihood (ML) method (substitutions type: nucleotide; model: Jukes–Cantor model). Green and blue triangles highlight *Heterobasidion irregulare*‐like and *Heterobasidion occidentale*‐like alleles from heterokaryotic isolates, respectively. Homokaryotic isolates from spores were represented by black circles. Bootstrap values are also shown

*EF‐1α* and *gpd* were chosen to build a tree using concatenated sequences. Only these two loci were chosen because the number of *RPB2* accessions to be used as references is rather limited in GenBank, and because the number of ITS alleles per isolates was >2, making the concatenation impossible. The concatenated *EF‐1α* and *gpd* tree defined two clusters, one comprising *H. irregulare* and one *H. occidentale*. As in the analysis of individual loci, several alleles of heterokaryotic isolates clustered in between these two clusters, were closer to *H. irregulare* and were considered as *H. irregulare*‐like, while others grouped with genotypes in the *H. occidentale* cluster and were considered as *H. occidentale*‐like (Figure 3). Alignments of all loci are deposited as FASTA files in the Dryad Digital Repository (https://doi.org/10.5061/dryad.6t7sh85). The hypothesis of equality of evolutionary rate as inferred by the Tajima's relative rate test was rejected for all *H. irregulare*‐like alleles, when compared with *H. irregulare* alleles (*p*‐value < 0.05). This was not true for the *H. occidentale*‐like alleles compared with *H. occidentale* sequences (*p*‐value > 0.05), with the exception of allele MH3011.a (*p*‐value 0.00389).

### 3.5 Multidimensional scaling, Bayesian, and PhyloNet analysis

**Figure 4:**
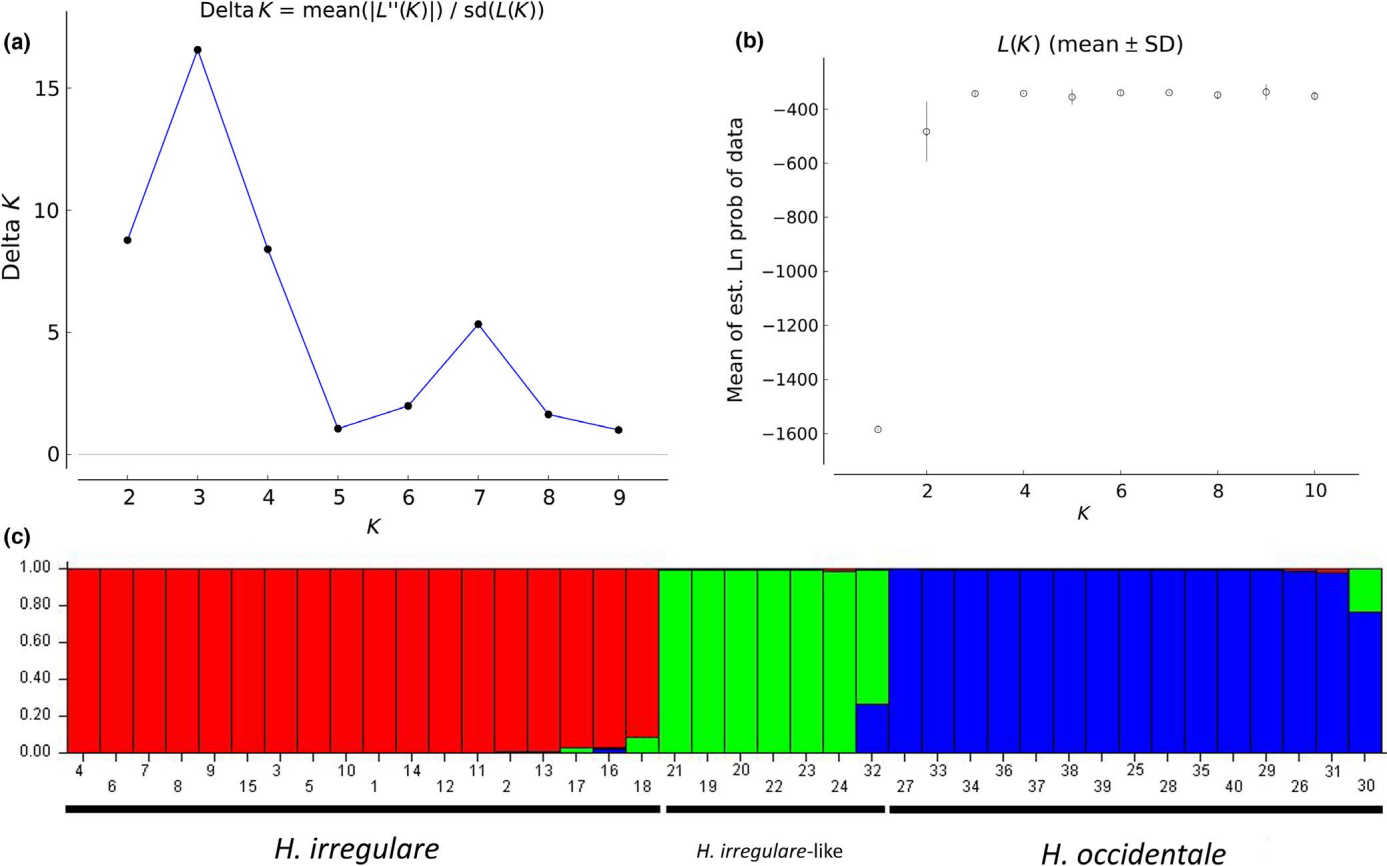
STRUCTURE analysis based on 687 sites of the concatenated sequences of *EF‐1α* and *gpd*. (a,b) Results of the Bayesian information criterion used to infer the number of genetic clusters, as generated by Structure Harvester. (c) Plot shows probability that concatenated alleles belongs to one of the three clusters, that is, *Heterobasidion irregulare* (red), *Heterobasidion occidentale* (blue), and *H. irregulare*‐like (green). Each concatenated allele is represented by a vertical line, which is partitioned into three colored segments that represent the estimated membership into one or the other cluster. Number from 1 to 18 represent *H. irregulare* allele (available in GenBank), from 19 to 24 represent *H. irregulare*‐like alleles of heterokaryotic isolates MH3001, MH3003, MH3005, MH3006, MH3007, and MH3011, from 25 to 30 represent *H. occidentale* alleles of heterokaryotic isolates MH3001, MH3003, MH3005, MH3006, MH3007, and MH3011, from 31 to 35 represent alleles from homokaryotic isolates II1A, III2B, II5A, II2B, II4C, and from 36 to 40 represent *H. occidentale* alleles (available in GenBank)

The PCoA on concatenated sequence alignments differentiated alleles from heterokaryotic larch isolates into either *H. occidentale*‐like or into *H. irregulare‐*like. While *H. occidentale*‐like alleles grouped close to *H. occidentale*, *H. irregulare*‐like alleles resulted as clearly distinct from *H. irregulare* alleles. Four out of five spore alleles fell into the *H. occidentale* group, while one (III2B) placed in between the *H. occidentale* and *H. irregulare*‐like groups, very close to *H. irregulare*‐like alleles (Figure S9). The result of the PcoA was confirmed by the Bayesian analysis. Based on STRUCTURE analysis, the optimal number of populations (*K*) as inferred by evaluating the Δ*K* was three (Figure 4). The three *K* included alleles clearly associated with three groups of haplotypes (concatenated alleles), namely *H. irregulare‐*, *H. occidentale‐*, and the *H. irregulare*‐like cluster. The Bayesian inference suggested that *H. irregulare*‐like alleles represent a genetic group clearly distinct from the other three (Figure 4).

**Figure 5:**
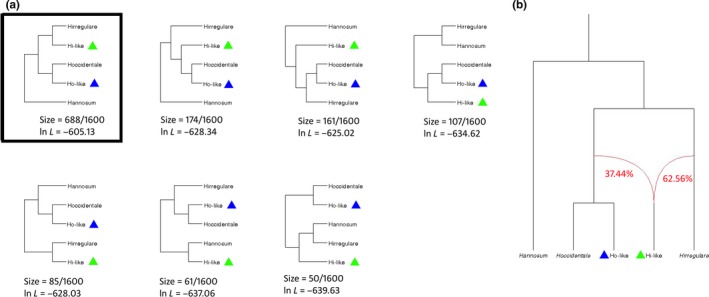
Results of the PhyloNet analysis. (a) The seven most likely tree topologies within the 95% credible set of network topologies that best fit with sequence data as inferred by PhyloNet. Likelihood scores (ln *L*) are visualized. Hi‐like (green triangles) represent *Heterobasidion irregulare*‐like alleles while Ho‐like (blue triangles) represent *Heterobasidion occidentale*‐like alleles. The optimal network is surrounded by a black square. (b) Reticulated (bifurcate) phylogenetic network as inferred by PhyloNet. Sequences of Hi‐like resulted to be hybrid between *H. occidentale*‐Ho cluster and *H. irregulare*. Branch lengths are not scaled to sequence divergence. The two inheritance probabilities, that is, parental contributions, for the reticulation edges (red lines) are also indicated

In order to test for evidence of hybridization events, PhyloNet analysis was carried out on concatenated sequences. Sixteen possible topologies of tree (95% credible set of topologies) out of 1,600 (the total number of possible trees generated by PhyloNet and subjected to the analysis) were inferred. Seven of them with sample size ≥50 (the number of times the topology is sampled based on PhyloNet analysis) were represented in Figure 5. The optimal reticulated network inferred by the software (with sample size = 688/1,600) showed that alleles of *H. occidentale*‐like clustered together with that of *H. occidentale*, while alleles of *H. irregulare*‐like were grouped with alleles of *H. irregulare* (Figure 5). Two reticulations were detected both involving the makeup of *H. irregulare*‐like alleles. One putative introgression was identified from the *H. occidentale* and *H. occidentale*‐like cluster to *H. irregulare*‐like alleles, and one from *H. irregulare* to *H. irregulare*‐like alleles, resulting in a bifurcate tree (Figure 5). Parental contributions to the allelic makeup of *H. irregulare*‐like alleles, expressed as inheritance probabilities, were 62.56% and 37.44% for the *H. irregulare* and the *H. occidentale* cluster, respectively.

## 4 DISCUSSION

### 4.1 *Heterobasidion* genotypes from Alpine larch are heterokaryotic hybrids harboring two different nuclei, but a single mitochondrial genome

Our survey of two distinct root disease centers in a high elevation mixed forest stand dominated by Alpine larch indicated that, in both instances, disease and mortality of Alpine larch were associated with infection by *Heterobasidion*. Unexpectedly, all fungal genotypes infecting larch were heterokaryotic (*n* + *n*) hybrids that included pairs of nuclei of different origin. These nuclei harbored novel and unreported nuclear alleles, but a comparison with known DNA sequences showed that in these hybrids, one nucleus possessed alleles that, albeit novel, could be clearly defined as being derived from an *H. occidentale* parent*,* while the other had novel alleles partially derived from an *H. irregulare* parent, but clearly distinct from published *H. irregulare* sequences. Sequence information indicated that each heterokaryotic genotype was a different haplotype, and thus, each represented the result of independent interspecific mating events. All genotypes collected during the survey, whether from wood, fruiting bodies, or trapped from the air, were characterized by an identical *atp6* sequence typical of the *H. occidentale* mitochondrion. This information, combined with the dominance of *H. occidentale* spores collected from the air, suggests *H. occidentale* is the dominant species at that site. This was to be expected, given that *H. occidentale* is normally associated with tree species growing both in the montane and Alpine ranges and is almost ubiquitous in true fir (*Abies* spp.) stands (Otrosina & Garbelotto, 2010), while *H. irregulare*, despite showing a partial habitat overlap with *H. occidentale*, is mostly associated with pines found at montane or lower altitudes. Additionally, mitochondria have been associated with host specificity; thus, they are likely to provide an adaptive fitness when infecting a specific host (Olson & Stenlid, 2001), and hence, the *H. occidentale* mitochondrion in genotypes with admixed nuclear content may favor infection of larches.

Despite its dominance, pure *H. occidentale* was not found to be infecting larches, suggesting three things: (a) Primary infection may be effected by *H. occidentale*, as suggested by the absolute dominance of *H. occidentale* mitochondrial *atp6* sequences, but *H. occidentale* mycelium later may be fertilized by *H. irregulare* spores or mycelium; (b) Alpine larch may be a universal host for both *H. occidentale* and *H. irregulare*, as suggested for European larch with regard to European *Heterobasidion* species (Garbelotto & Gonthier, 2013), thus providing the necessary habitat for interspecific mating to occur, and (c) Hybrid genotypes may be at an advantage when colonizing larch.

Although further research needs to confirm the three hypotheses above, the hybrid genotypes we collected provided us with a unique opportunity to study the molecular evolution of interspecific hybrids. Ecological partitioning between the two *Heterobasidion* species, and the abundance of subalpine fir (a *H. occidentale* host) in and immediately below the study sites, suggest only *H. occidentale* should be present at high altitudes (Otrosina & Garbelotto, 2010). Our air sampling confirmed that expectation. On the other hand, *H. irregulare* may have arrived at such high altitude because of an increase in its population size due to the relative recent increased logging of ponderosa pine at lower altitudes in the Bitterroot Mountain Range (Lockman, 2006). At the study site, *H. irregulare* seems to be particularly rare, as expected of a population that is not native to the site, but may have reached it thanks to the sporadic effects of ascending air currents. However, our trapping produced only four *H. occidentale* spores (corresponding to 14 spores/m^2^ h) suggesting populations of *H. occidentale* may be small, probably because the site is at the altitudinal limit for the survival of this fungal species. Numbers of spores trapped in other studies using comparable sampling approaches are significantly higher than the number reported here (Gonthier et al., 2005, 2007; Gonthier, Lione, Giordano, & Garbelotto, 2012). Hence, hybridization may have been favored by demographic conditions, for example, by low numbers of individuals of both parental species increasing the chances of interspecific encounters and mating (Seehausen, 2004).

### 4.2 Nuclei of hybrid isolates had either *H. occidentale* alleles or alleles derived from both *H. irregulare* and *H. occidentale*

Sequences of four nuclear loci indicated that allelic variation was large in hybrids and most sequences were not a perfect match for pure *H. occidentale* or pure *H. irregulare* GenBank sequences. This suggested that the hybrid genotypes were not F1 hybrids as originally thought (Lockman et al., 2014), but rather they may represent the progeny of hybrids backcrossed with other genotypes. Flow cytometry results confirmed this observation and showed that none of the genotypes from wood or from fruiting bodies were diploid, as it would have been expected for first‐generation *Heterobasidion* hybrids (Garbelotto et al., 2004).

Large allelic variation was identified in hybrid genotypes: most nuclear alleles were novels, indicating hybridization enhanced the genetic variability in a population. All alleles were identifiable as being derived either from *H. occidentale* or from *H. irregulare* parental populations; however, our results indicated a striking difference between variation levels in *H. occidentale‐*like alleles and variation levels in *H. irregulare*‐like alleles. *Heterobasidion occidentale‐*like alleles, albeit novel, could not be distinguished from other *H. occidentale* alleles known for North America and thus the nuclei that bear them should be referred to simply as *H. occidentale*. Conversely, *H. irregulare*‐like alleles were highly divergent from *H. irregulare* alleles. Although the level of sequence polymorphisms found in *H. irregulare‐*like nuclei is such to presume a possible third parental species may have been involved in the hybridization process, we discarded this hypothesis because, when analyzing *H. irregulare*‐like sequences: (a) *gpd* and *RPB2* alleles clearly belong to either *H. irregulare* or *H. occidentale*, and alleles of a putative third species were absent; (b) no sequence of a third mitochondrial genome has been found in this or any other studies from North America; and (c) the high ITS and *EF1‐α* sequence divergence compared to published *H. occidentale* and *H. irregulare* sequences can be ascribed to admixing caused by hybridization, as shown by evidence of loss of concerted evolution in the case of all ITS alleles (see section below), and by the results of the PhyloNet analysis in the case of *EF1‐α H. irregulare‐*like alleles.

All of the SNPs in *EF1‐α* are synonymous with either *H. irregulare* or *H. occidentale* sequences, and PhyloNet identified the novel *H. irregulare*‐like alleles as the result of admixing of both parental alleles. This finding demonstrates that all *EF1‐α* alleles analyzed in this study were true *Heterobasidion* sequences, and not transversally inherited EF‐like (EFL) GTPases, like those occasionally documented for other organisms (Keeling & Inagaki, 2004). Moreover, the presence of EFLs has never been documented in *Heterobasidion* genomes (Olson et al., 2012; Sillo, Garbelotto, Friedman, & Gonthier, 2015). Our findings strongly support the hypothesis that, although *H. irregulare*‐like alleles may have reached a threshold of sequence divergence in line with that expected of a new taxonomic entity distinct from both *H. irregulare* and *H. occidentale*, they are not derived from a hypothetical third taxonomic entity. As such, nuclei‐bearing *H. irregulare*‐like alleles should be more properly identified as *H. irregulare* × *occidentale*, due to their hybrid origin.

### 4.3 Loss of concerted evolution and presence of intermediate alleles between *H. irregulare* and *H. occidentale* suggest that hybridization processes are ongoing

The loss of concerted evolution of ITS copies (nrDNA) of both *H. irregulare*‐like and *H. occidentale*‐like alleles might be a footprint of rampant ongoing hybridization (Muir, Fleming, & Schlötterer, 2001; Xu, Zeng, Gao, Jin, & Zhang, 2017). After hybridization processes, recombination of ITS regions followed by homogenization of repeats generally occurs, as observed in several basidiomycetous fungi (Hughes & Petersen, 2001; Kauserud, Svegården, Decock, & Hallenberg, 2007). In the current study, the retained heterogeneity of ITS copies regarded as an incomplete concerted evolution might suggest that the hybridization process is still ongoing (Buckler, Ippolito, & Holtsford, 1997). The presence of alleles with different matching identity with *H. irregulare* and *H. occidentale* in *gpd* and *RPB2* alleles might be also a footprint of a dynamic evolutionary process, such as hybridization. Results of Tajima's test confirmed that hybridization is affecting the evolutionary rates differently in the two parental nuclei. The test for equality of evolutionary rate in fact demonstrated that *H. irregulare*‐like alleles did not follow the classical molecular clock hypothesis, but instead they seem to have evolved more rapidly than *H. occidentale*‐like alleles in hybrid isolates.

**Figure 6:**
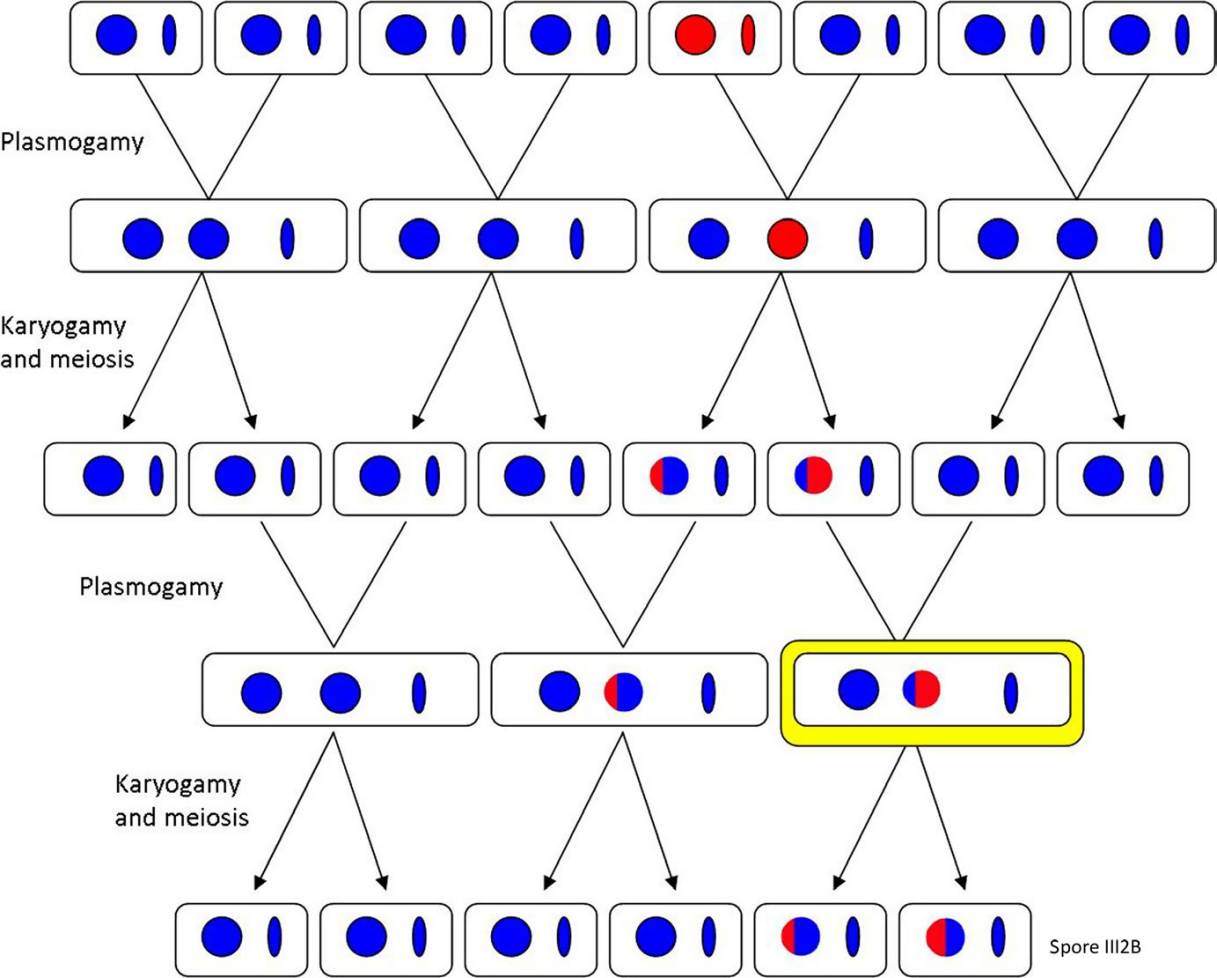
Schematic representation of the proposed evolutionary scenario. Circles represent nuclei, and ellipses represent mitochondria. Homokaryotic (*n*) isolates from *Heterobasidion occidentale* (blue) and *Heterobasidion irregulare* (red) mated through plasmogamy resulting in first‐generation heterokaryotic (*n* + *n*) isolates. After karyogamy and meiosis in the fruit body, haploid (*n*) spores were released and mated again through plasmogamy resulting in a second generation of hybrid heterokaryotic isolates. Heterokaryotic hybrids between *H. occidentale* and *H. irregulare* generated spores with haploid nuclei characterized by different percentages of admixture between the two parental species: the repeated backcrossing of admixed genotypes with pure *H. occidentale* isolates resulted in a loss of *H. irregulare* genetic inheritance. The current situation, highlighted in yellow, is characterized by heterokaryotic isolates affecting larches harboring one *H. occidentale* nucleus and one nucleus resulting from the genomic admixture between *H. irregulare* and *H. occidentale*. Mitochondria of *H. occidentale* are dominant probably because of the unilateral backcrossing of admixed genotypes solely with *H. occidentale*, and possibly because of a fitness advantage in larches conveyed by the *H. occidentale* mitochondrial genome. In this scenario, the haploid isolate III2B (*n*) may be the result of meiosis occurring in a hybrid fungal fruit body

Interestingly, *H. irregulare*‐like *EF1‐α* and ITS alleles both harbored unique SNPs that may be the result of hybridization‐mediated evolution. We postulate that the generation of a novel *Heterobasidion* nucleus can be ascribed to backcrossing continuously occurring only between hybrids and *H. occidentale* individuals, due to the local dominance of *H. occidentale* combined with the local rarity, or even possibly the absence, of a viable local *H. irregulare* population (Figure 6). Thus, at each backcrossing event, the nuclear content of *H. occidentale* may increase in the *H. irregulare* nuclear genome, causing a further divergence from the original parental population (Figure 6). Additionally, the constant obligate interaction between the *H. irregulare* nucleus and the *H. occidentale* mitochondrion may have facilitated this evolutionary process by adaptive necessity. This is consistent with a recent study demonstrating that mitonuclear interactions in *Heterobasidion* spp. may play a role in shaping the nuclear genome (Giordano, Sillo, Garbelotto, & Gonthier, 2018). Rapid evolution and speciation by hybridization have been described in detail for plants, animals, fungi, and oomycetes (Gross & Rieseberg, 2005; Mallet, 2007; Schumer, Rosenthal, & Andolfatto, 2014). The same is true for unidirectional introgression; in most of the literature, the unidirectionality of introgression has been ascribed to female infertility or significant malaptation of crosses in the other direction (Field, Ayre, Whelan, & Young, 2011; Takahashi et al., 2017; Tiffin, Olson, & Moyle, 2001). Because of the absence of male and female individuals in basidiomycetous fungi (Heitman, Sun, & James, 2013), it is unlikely that paternal or maternal factors may explain the unidirectionality of introgression in the *Heterobasidion* hybrids found on Alpine larches. Rather, we believe the primary cause for the repeated backcrossing of hybrid progeny into *H. occidentale* may be due to the extremely low abundance of *H. irregulare* individuals. This unidirectionality may have been somewhat reinforced by a significant greater adaptation of *H. occidentale* and of the *H. occidentale* mitochondrion when infecting larch and/or in order to survive at extremely high altitudes. It has been documented that the transfer of genetic material between hybridizing species through backcrossing with one or both parental species might play a crucial role in the fast adaptation of hybrids to new environments (Arnold, 2004; Gladieux et al., 2014).

Although it cannot be excluded that different three dimensional folding of novel alleles during transcription may favor their selection (Chothia & Finkelstein, 1990), all of the novel alleles identified in this study were synonymous to ones previously identified in parental populations. This further reinforces the argument that the primary driver in the hybridization‐mediated evolution acting on *H. irregulare* nuclei was mostly a consequence of its repeated recombination with *H. occidentale*. The creation of new alleles through intralocus recombination reported in this work has also been documented in *Heterobasidion annosum* × *H. irregulare* and *Flammulina* hybrids (Gonthier & Garbelotto, 2011; Hughes & Petersen, 2001). We believe this to be one of the first studies reporting hybridization‐mediated evolution of one of the two parental nuclei due to unbalanced presence of parental individuals. We suggest that evolution of alleles in the *H. occidentale* nucleus was less evident because of the unidirectional backcrossing between hybrids and pure *H. occidentale* (Figure 6).

### 4.4 Hybridization‐mediated evolution resulted in a novel taxonomic entity associated with Alpine larch in Montana, but with potentially far‐reaching consequences

The fact that one of the nuclei in heterokaryotic hybrids has evolved to be clearly distinct from its progenitors makes this an irreversible evolutionary process apparently associated with infection of Alpine larch by what may be a novel *Heterobasidion* taxon. Given the breadth of previous surveys on this genus, it is likely the distribution of this novel taxonomic entity may be somehow limited, maybe in association with high elevation Alpine larches. Although the distribution of hybrids may be limited, unidirectional introgression of alleles from the *H. irregulare*‐like nuclei into *H. occidentale* may not be limited, especially for those alleles that may confer an adaptive advantage to the recipient species (Currat, Ruedi, Petit, & Excoffier, 2008). An introgression of *H. irregulare* alleles into *H. occidentale* may allow the latter species to thrive at lower altitudes and to coinfect some of the *H. irregulare* hosts. This adaptation could result in a significant negative impact on forest ecosystems that may have to face a new pathogenic entity possibly combining virulence characters of both parental species. Disease symptoms in larch included both a heartrot similar to that caused by *H. occidental*e, and necroses in the cambial layer similar to those created by *H. irregulare*, suggesting a mixed disease ecology of genotypes with admixed genomes. The presence of interspecific hybrids on Alpine larch in the absence of stumps may also provide one explanation for the horizontal gene transfer documented to have occurred in the past between the two North American species of *Heterobasidion* (Garbelotto et al., 1996; Linzer et al., 2008).

## 5 CONCLUSIONS

It is now widely recognized that hybridization is an important evolutionary process and may play a crucial role in speciation (Gross & Rieseberg, 2005; Mallet, 2007; Schumer et al., 2014; Taylor & Larson, 2019). The molecular characterization of *H. irregular* × *H. occidentale* hybrids presented in this study suggests that an anthropogenic disturbance may have lead to the hybridization‐mediated evolution of a novel hybrid pathogen affecting Alpine larch. Furthermore, hybridization appears to have disproportionately affected the evolution of alleles of one of the two parents, and a higher rate of evolution was detected for the more rare parental species.

A broader sampling in the area of Bitterroot Mountains is necessary to monitor the range and the impacts on Alpine larch of the hybrid taxon here described. In addition, a large‐scale genomic analysis will be pivotal to detect which chromosomal blocks and genes may be subjected to higher recombination and evolution levels, in order to link genomic and ecological traits of hybrids.

## CONFLICT OF INTEREST

None declared.

## AUTHOR CONTRIBUTIONS

MG and BL performed the fieldwork. MG and FS performed DNA amplification, allelic cloning, and sequencing. FS performed molecular analyses. FS, MG, and PG critically reviewed the manuscript. TK performed flow cytometry. FS and MG wrote the manuscript. All authors critically reviewed and edited the draft.

## Data Availability

All data used in this study are included in the paper and in the Appendix, and deposited in Dryad Digital Repository (https://doi.org/10.5061/dryad.6t7sh85; https://datadryad.org/).
