# Supplementary material for: Molecular analyses identify hybridization‐mediated nuclear evolution in newly discovered fungal hybrids
Source: Ecol Evol. 2019 May 9;9(11):6588–605. doi: 10.1002/ece3.5238 (PMC6580273; doi:10.1002/ece3.5238)
Supplement: Supplementary file 1 [file ECE3-9-6588-s001.doc]

**Appendix**

**Molecular analyses identify hybridization-mediated nuclear evolution in newly discovered fungal hybrids**

Fabiano Sillo^1^, Paolo Gonthier^1^, Blakey Lockman^2^, Takao Kasuga^3^, and Matteo Garbelotto^1^,^4^

**Author affiliations**:

^1^University of Torino, Department of Agricultural, Forest and Food Sciences (DISAFA), Largo Paolo Braccini 2, I-10095 Grugliasco (TO), Italy

^2^USDA Forest Service, Pacific Northwest Region, State and Private Forestry, Portland, Oregon, USA

^3^ USDA Agricultural Research Service, Crops Pathology and Genetics Research Unit, 95616 Davis, California, USA

^4^University of California, Berkeley, Department of Environmental Science, Policy and Management, Forest Pathology and Mycology Laboratory, 54 Mulford Hall, 94720 Berkeley, California, USA

Corresponding Author: **Matteo Garbelotto**, matteog@berkeley.edu

**Supplementary tables**

Table S1……………………………………………………………………………...…………...3

**Supplementary figures**

Figure S1……………………………………………………………………………...…………...4

Figure S2…………………………………………………………………………...……………...5

Figure S3………………………………………………………………………...………………...6

Figure S4……………………………………………………………………...…………………...7

Figure S5………………………………………………………………………...………………...8

Figure S6………………………………………………………………………...………………...9

Figure S7………………………………………………………………………...……………….10

Figure S8………………………………………………………………………...……………….11

Figure S9………………………………………………………………………...……………….12

**Table S1.** Source species and GenBank accession number of sequences used for the concatenated phylogenetic tree showed in Figure 3.

| **Isolate ID#** | **Locus** | |
| --- | --- | --- |
|  | ***gpd*** | ***EF-1α*** |
| *H. occidentale* PFC 5357 | KP863649.1 | KP863613.1 |
| *H. occidentale* Bbe | EU190181.1 | EU190074.1 |
| *H. occidentale* PFC 5282 | KP863638.1 | KC571672.1 |
| *H. occidentale* MON108 | FJ627497.1 | FJ627416.1 |
| *H. occidentale* MON109 | FJ627703.1 | FJ627498.1 |
| *H. irregulare* Conk3 | EU190125.1 | EU190014.1 |
| *H. irregulare* 1116-1 | EU190111.1 | EU190001.1 |
| *H. irregulare* 24086C | EU190115.1 | EU190005.1 |
| *H. irregulare* Conk1 | DQ916101.1 | DQ916087.1 |
| *H. irregulare* 11063B | EU190112.1 | EU190002.1 |
| *H. irregulare* 94-190 | EU190121.1 | EU190011.1 |
| *H. irregulare* CL-6 | EU190123.1 | EU190013.1 |
| *H. irregulare* 12023B-2 | EU190114.1 | EU190004.1 |
| *H. irregulare* 4-2-1 | EU190107.1 | EU189996.1 |
| *H. irregulare* REU120 | EU190144.1 | EU190036.1 |
| *H. irregulare* MON111 | FJ627499.1 | FJ627418.1 |
| *H. irregulare* Bbb | EU190143.1 | EU190031.1 |
| *H. irregulare* c4 | EU190140.1 | EU190028.1 |
| *H. irregulare* T338 | EU190134.1 | EU190023.1 |
| *H. irregulare* MON112 | FJ627500.1 | FJ627418.1 |
| *H. irregulare* MON114 | FJ627502.1 | FJ627418.1 |
| *H. irregulare* MON113 | FJ627501.1 | FJ627418.1 |
| *H. irregulare* 398-2 | EU190133.1 | EU190022.1 |
| *H. annosum* 00-071/1 | EU190162.1 | EU190054.1 |
| *H. annosum* 00-070/1 | EU190161.1 | EU190053.1 |
| *H. annosum* 417P | EU190149.1 | EU190042.1 |

**Figure S1.** Estimation of nuclear content of *Heterobasidion* spp. by means of flow cytometry using *A. fumigatus* and *A. thaliana* as internal size standards.


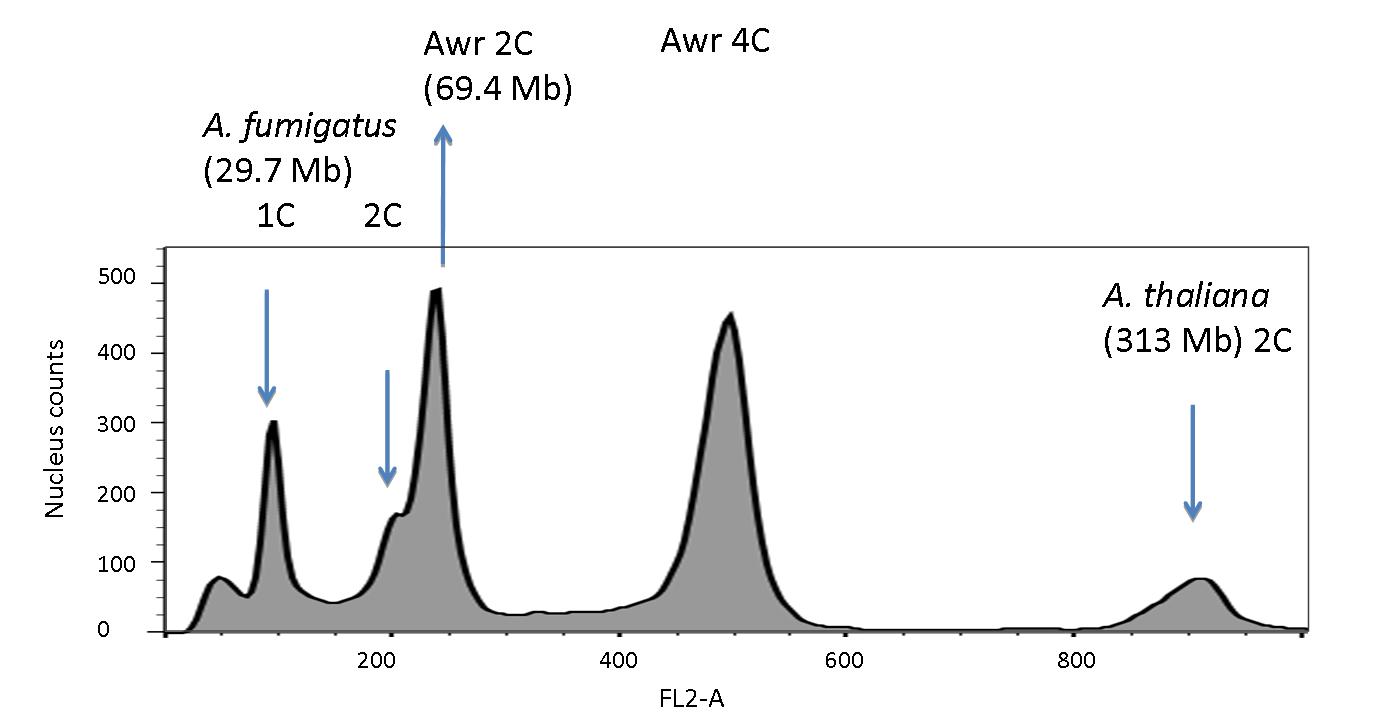


**Figure S2.** Alignment of *gpd* alleles (see Table 2) of heterokaryotic isolates against *H. irregulare* and *H. occidentale* *gpd* consensus sequences. Polymorphisms highlighted in shades of red represent SNPs related to *H. irregulare*, while those in shades of blue represent SNPs related to *H. occidentale*. Nucleotides written in lowercase represent bases assigned after extraction of homozygotic alleles from heterozygotic sequences.

**
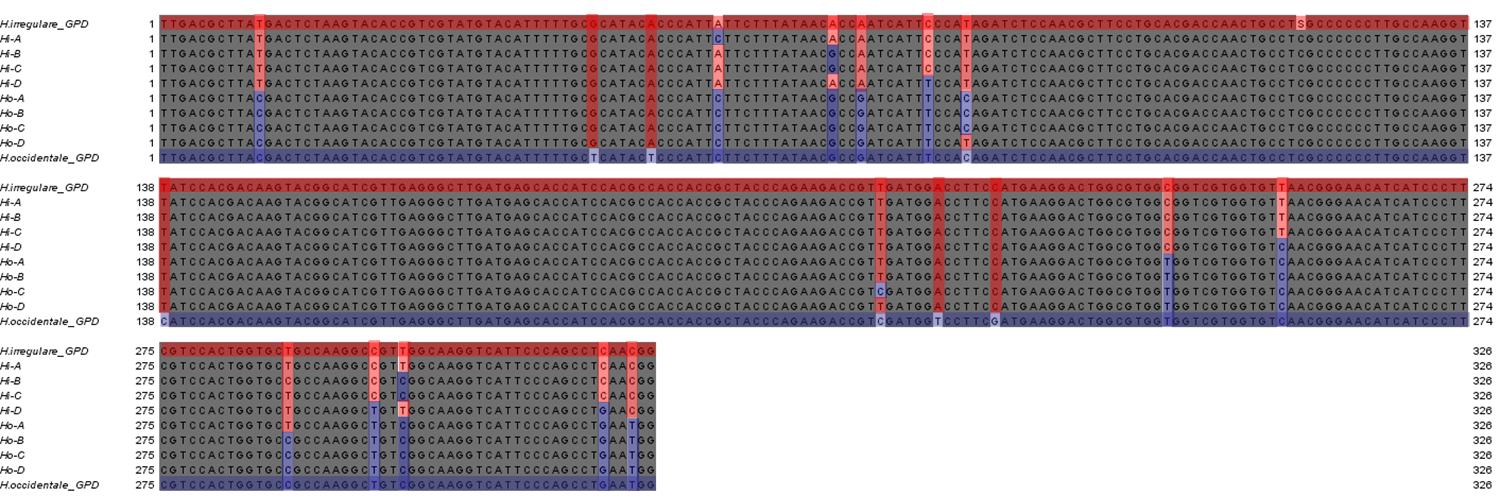
**

**Figure S3.** Alignment of *RPB2* alleles (see Table 2) of heterokaryotic isolates against *H. irregulare* and *H. occidentale* gpd consensus sequences. Polymorphisms highlighted in shades of red represent SNPs related to *H. irregulare*, while those in shades of blue represent SNPs related to *H. occidentale*. Nucleotides written in lowercase represent bases assigned after extraction of homozygotic alleles from heterozygotic sequences.

**
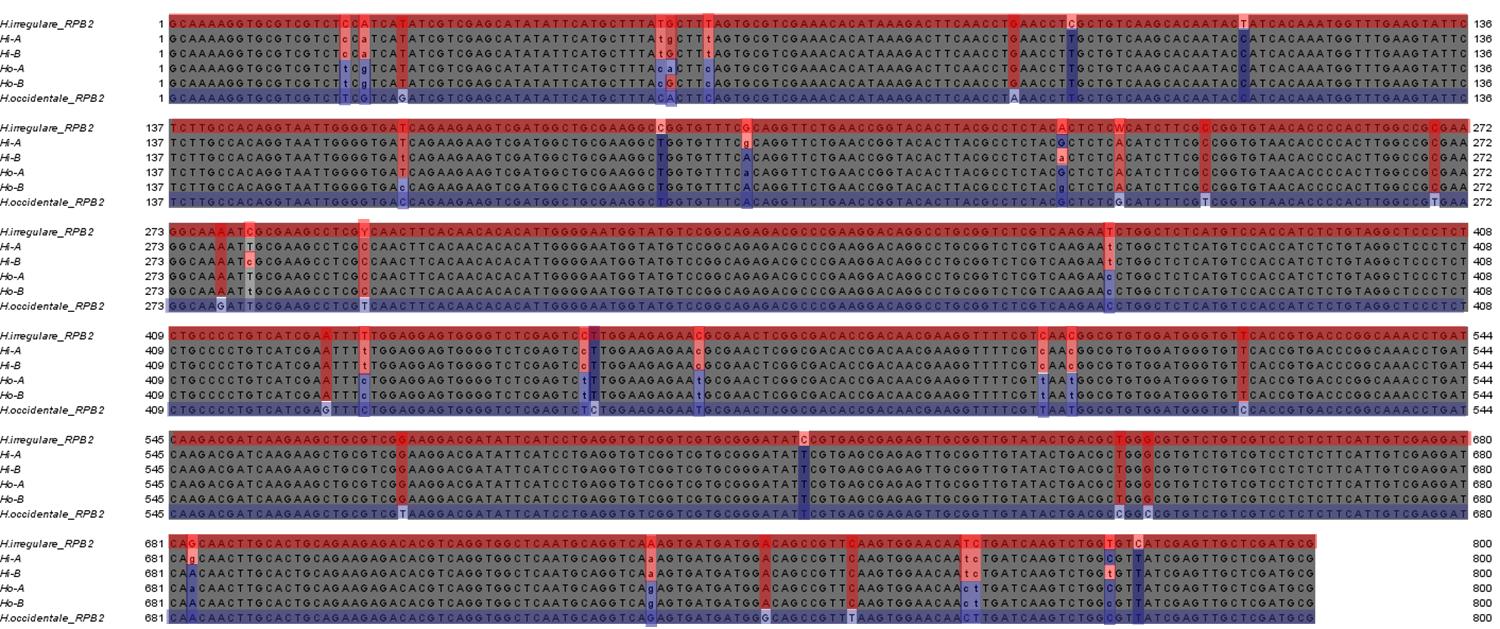
**

**Figure S4.** Phylogenetic Maximum Likehood (ML) tree of *atp6* sequences. Sequences of heterokaryotic (ploidy = *n+n*) isolates are represented by filled triangles (sequences coming from the same isolate are visualized as triangle with the same shape and colour). Sequences of homokaryotic (ploidy = n) isolates from spores are represented by black filled circles. Bootstrap values are also shown.

**Figure S5.** Phylogenetic ML tree of *gpd* sequences. Heterozygotic sequences inclusive of SNP ambiguities of heterokaryotic (ploidy = *n+n*) isolates were used in this analysis, and are represented by filled triangles (sequences coming from the same isolate are visualized as triangle with the same shape and colour). Homozygotic sequences of homokaryotic (ploidy = *n*) isolates from spores are represented by black filled circles. Bootstrap values are also shown.

**Figure S6.** Phylogenetic ML tree of *RPB2* sequences. Heterozygotic sequences inclusive of SNP ambiguities of heterokaryotic (ploidy = *n+n*) isolates were used in this analysis, and are represented by filled triangles (sequences coming from the same isolate are visualized as triangle with the same shape and colour). Homozygotic sequences of homokaryotic (ploidy = *n*) isolates from spores are represented by black filled circles. Bootstrap values are also shown.

**Figure S7.** Phylogenetic ML tree of *EF-1α* cloned sequences. Sequences of heterokaryoric (ploidy = *n+n*) isolates are represented by filled triangles (sequences coming from the same isolate are visualized as triangle with the same shape and colour). Sequences of homokaryotic (ploidy = *n*) isolates from spores are represented by black filled circles. Bootstrap values are also shown.

**Figure S8.** Phylogenetic ML tree of ITS cloned sequences. Cloned homozygotic sequences of heterokaryotic isolates (ploidy = *n+ n*) are represented by filled triangles (sequences coming from the same isolate are visualized as triangle with the same shape and colour). Homozygotic sequences of homokaryotic (ploidy *n*) isolates from spores are represented by black filled circles. Bootstrap values are also shown.

**Figure S9.** PCoA based on distance matrix within concatenated sequences of *Heterobasidion* spp. Green and blue triangles represent *H. irregulare-*like and *H. occidentale-*like concatenated alleles of heterokaryotic isolates, respectively, while black filled circles represent concatenated alleles of homokaryotic isolates (from spores), all generated in this study. Red triangles represent WA *H. irregulare* isolates, orange triangles represent EA *H. irregulare* isolates, brown triagle represents a Mexican *H. irregulare* isolate, blue circles represent *H. occidentale* and yellow triangle represent European *H. annosum* s.s. isolates. Percentages of variance explained by PC1 (x-axis) and PC2 (y-axis) are also showed.

**
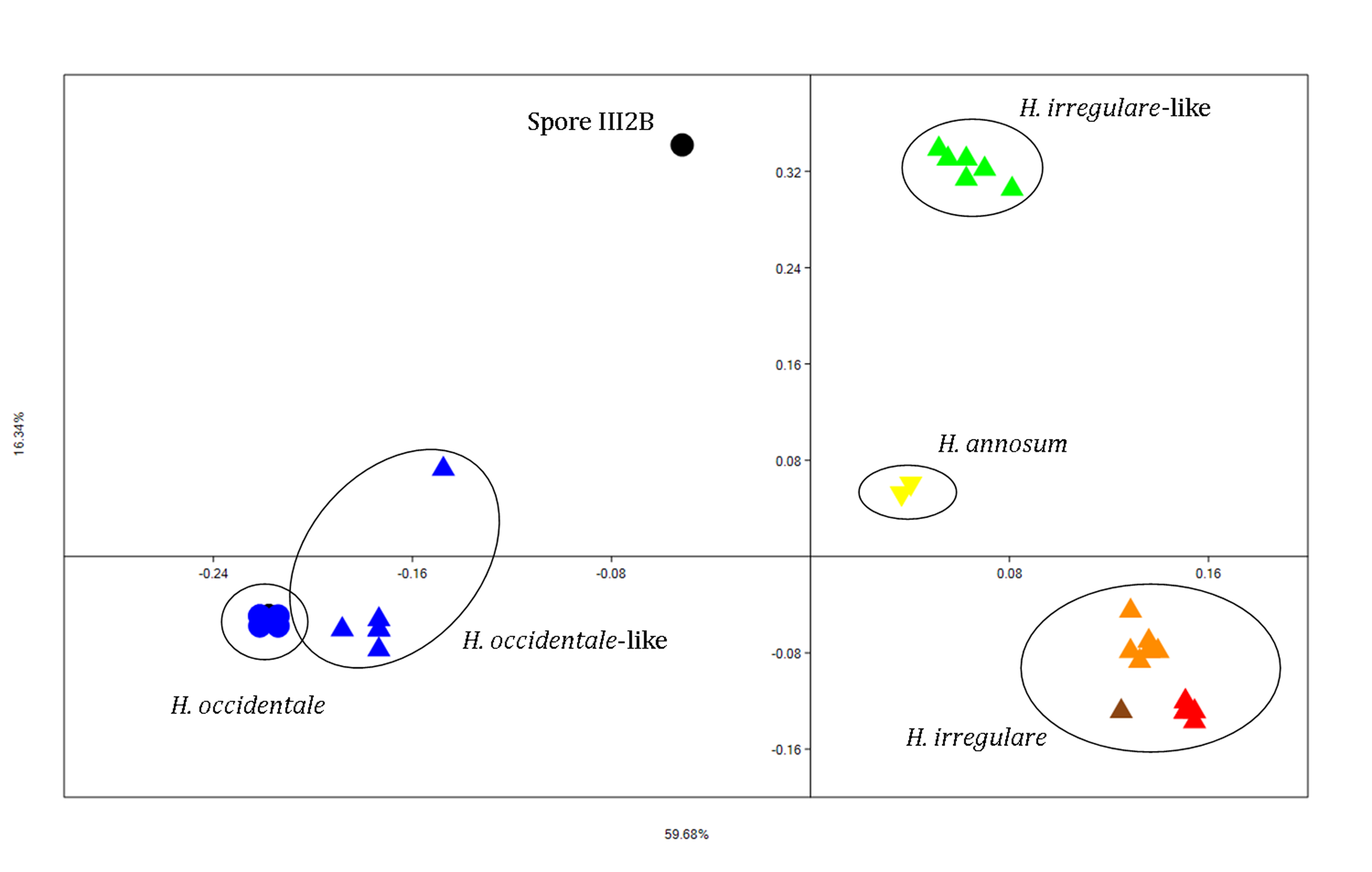
**
